# National

**Published:** 1896-11

**Authors:** 


### CONTROL WORK.

---

*National.* The Acting Secretary of Agriculture has issued a notice that cattle infected with Southern cattle-ticks disseminate Texas fever, and that, under the laws relating to the control of contagious and infectious diseases of animals, the regulations of the Bureau of Animal Industry, dated February 1, 1896, are hereby amended by an additional section as follows: "Cattle originating outside of the district described by the order dated February 1, 1896, as amended by subsequent orders, and which are infected with the *boophilus bovis* ticks, shall be considered as infectious cattle, and shall be subject to the rules and regulations governing the movement of Southern cattle. Stock-yard companies receiving such cattle shall place the same in the pens set aside for the use of Southern cattle, and transportation companies are required to clean and disinfect all cars and vessels which have contained the same, according to the requirements of this department."

Hog-cholera is prevalent in many of the Western States, especially so in Ohio, Indiana, and South Dakota.

